# Biological studies of clavine alkaloids targeting CNS receptors

**DOI:** 10.3389/fpsyt.2023.1286941

**Published:** 2023-11-21

**Authors:** Nikhil R. Tasker, Ethan J. Pazur, Peter Wipf

**Affiliations:** Department of Chemistry, University of Pittsburgh, Pittsburgh, PA, United States

**Keywords:** psychedelic therapies, neuropsychiatric diseases, anxiety, depression, lysergol, cycloclavine, agroclavine, ergot alkaloids

## Abstract

In contrast to well established psychedelics such as lysergic acid diethylamide (LSD) and psilocybin, ergot alkaloids of the clavine subclass have not been thoroughly investigated, in spite of their broad occurrence in nature and their well-established potent physiological effects. This study presents the current knowledge on the biological properties of clavine alkaloids, draws comparisons to the pharmacology of ergolines and related psychedelics, and demonstrates opportunities to develop novel structure–activity relationship (SAR) profiles. The latter could usher in a new stage of medicinal chemistry studies that enable an expansion of the currently structurally limited portfolio of psychedelic therapeutics.

## 1 Introduction

Psychedelic compounds have been used by a variety of cultures for centuries due to their ability to elicit an altered state of consciousness, evoke transcendence, reopen the social reward learning critical period, and to heal a variety of health disorders (1). Traditionally used by communities in South America and Africa for religious ceremonies and rituals, these compounds share a potential for substance abuse but are now re-emerging as potential therapeutics in North America and Europe (2). The demand for psychedelic therapy (3) and psychedelic wellness retreats (4, 5) has seen tremendous growth in recent years, in part driven by microdosing applications and the combination with traditional psychotherapeutic counseling. Users experience an altered state that involves changes in sensory perception and mood and may undergo dissociation or a loss of self-identity (6–10). An ability to reset a social reward learning mechanism might be a shared feature across psychedelic drugs (11). Interestingly, some users claim that a psychedelic experience permanently changed their outlook on life and/or improved their quality of life long after the experience (12). Although these claims are subjective, there is increasing evidence that suggests psychedelics can be used to treat psychiatric diseases, including anxiety and major depressive disorders (13, 14).

**Figure 1:**
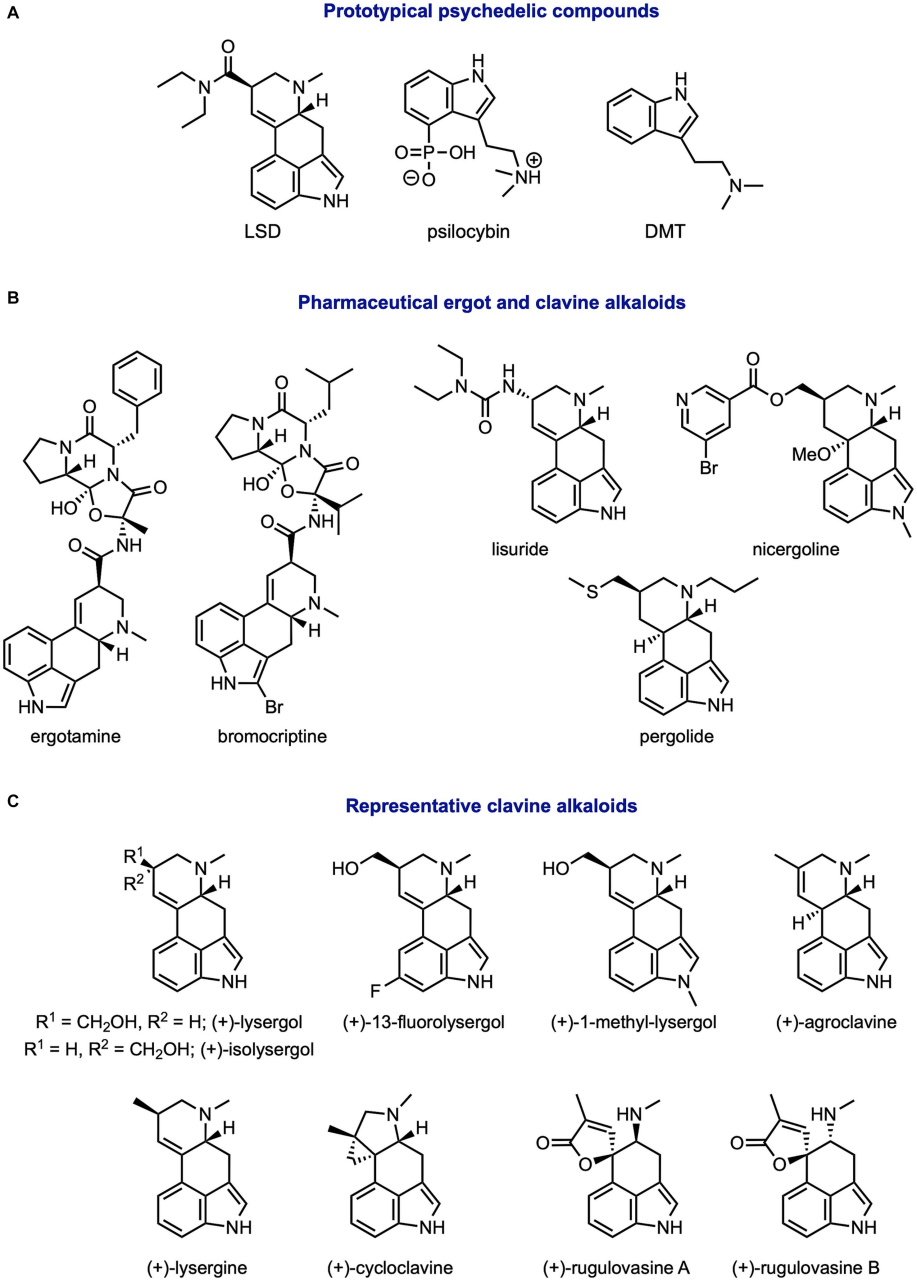
Structures of common ergot and clavine alkaloids. **(A)** Prototypical psychedelics, **(B)** pharmaceutical alkaloids, and **(C)** other studied clavine alkaloids.

Historically, scientific research into psychedelics and their therapeutic benefits has been slow and controversial. Classic psychedelics including lysergic acid diethylamide (LSD), psilocybin, dimethyltryptamine (DMT), and mescaline were used in the 1950s and 60s to treat alcoholism, addiction, anxiety, and trauma (Figure 1) (15, 16). LSD was also used to obtain a model for psychosis and schizophrenia due to its ability to elicit symptoms of these disorders at high doses (17–19). Unfortunately, there were growing concerns of abuse regarding psychedelics and these compounds became associated with counterculture. As a result, the Drug Abuse Control Amendments of 1965 and the Controlled Substances Act of 1970 were passed. These policies severely limited the availability of psychedelic compounds for researchers and clinical investigations into these substances were stalled. Twenty years later, many seminal and novel studies on psychedelic compounds emerged. Among the earliest examples are Rick Strassman’s studies on the dose dependent effects of DMT in humans (20, 21). These promising psychiatric and clinical investigations of psychedelics have brought these compounds out of the dark ages (22). Indeed, this new wave of vibrant research into psychedelic compounds has ushered in a “psychedelic renaissance.” (23).

Considerable efforts have been expended to elucidate how psychedelics and structurally related compounds elicit their unique biological responses. LSD, an ergot alkaloid and a prototypical psychedelic, has strong interactions with aminergic G-protein-coupled receptors (GPCRs), including serotonin, dopamine, histamine, adrenergic and muscarinic receptors (24–30). Recently, Lewis et al. screened LSD at 33 human aminergic GPCRs using a G protein dissociation BRET-based assay as part of a pharmacological profiling of a non-hallucinogenic LSD analog (*vide infra*) (31). The receptors were ranked by calculating a relative activity (log E_MAX_/EC_50_), and the top-ranked GPCRs included all five of the 5-HT_1_ and the three 5-HT_2_ subtypes, 5-HT_6_, and the α_2C_ adrenergic receptor. Dopamine receptors D_2–4_ and the human serotonin receptors 5-HT_5A_ and 5-HT_7A_ were among the top 15 hits. LSD showed pan-agonism at 5-HT_1_ (EC_50_ = 0.62–2.38 nM) and 5-HT_2_ receptors (EC_50_ = 0.30–0.66 nM). At the remaining serotonin receptors, LSD demonstrated potent agonism at 5-HT_6_ (EC_50_ = 0.13 nM), antagonism at 5-HT_7A_, and only partial agonism at 5-HT_5A_ (EC_50_ = 2.33 nM, E_MAX_ = 11% relative to 5-HT). LSD was an agonist at the D_2_, and D_4_ receptors (EC_50_ = 2.17 and 4.03 nM, respectively) and a partial agonist at D_3_ (EC_50_ = 7.57 nM, E_MAX_ = 75% relative to dopamine). Finally, LSD showed partial agonism at α_2C_ (EC_50_ = 0.56 nM, E_MAX_ = 80% relative to norepinephrine). Ergotamine is a natural ergot alkaloid and a precursor of LSD with a similar pharmacological profile to LSD but is not generally considered as psychoactive. Ergotamine differs from LSD by its cyclic peptide moiety appended to the amide sidechain. Peng et al. profiled ergotamine’s pharmacology and discovered that ergotamine has a good affinity (*K_i_* < 10 μM) for ~70% of all human aminergic GPCRs (32). Furthermore, 15 of these receptors show low or sub-nanomolar affinity based on a radioligand competition binding assay. While ergotamine displayed agonism at 5-HT_2A–C_ and most 5-HT_1_ receptors, it did not display agonist activity at the D_3_, *α*_1B_, *α*_1D_, or *β*_2_ receptors despite high affinities, suggesting it acts as an antagonist at these receptors. Finally, ergotamine was an inverse agonist at 5-HT_7_, and further profiling showed that it also has opioid receptor agonist activity.

Most targets of LSD and/or ergotamine have been implicated in numerous diseases and disorders. Many 5-HT_1_ receptors show promise for the treatment of anxiety, depression, PTSD, and migraines (33–38). 5-HT_2A_ activation promotes neuronal growth and dendritic arbor complexity, and these outcomes are linked to improvements in mood and addiction disorders (39–41). The 5-HT_2C_ receptor contributes to mood regulation and is a common target for treating obsessive compulsive disorder (OCD) and the mode of action of antipsychotics (42–46). This mechanism has also been implicated in treating addiction and obesity (47). Targeting 5-HT_6_ has been shown to improve learning and memory and may also treat cognitive impairment (48–50). Additionally, 5-HT_7_ receptors regulate inflammation across the body, and agonists are associated with anti-inflammatory and neuroprotective effects (51–53). Finally, dopamine agonists are commonly used for treating involuntary movement disorders (54–56). However, activation of 5-HT_2A_ is also believed to be a primary mechanism in which psychedelics produce their psychoactive effects (57–63). In contrast, activation of 5-HT_2B_ is associated with cardiac valvulopathy, and thus targeting this receptor is generally undesirable (64, 65). Therefore, many studies aim to discover psychedelic analogs that are selective for other receptors over 5-HT_2A/2B_ or demonstrate functional selectivity, in which a ligand stabilizes distinct receptor conformations that can selectively activate or deactivate downstream signaling pathways while keeping others unchanged (66, 67).

Biochemical, structural biology, bioengineering, and docking studies have all aided in the pursuit of non-hallucinogenic psychedelic analogs by pinpointing key structural features of compounds that elicit biased agonism. In 2013, Wacker et al. demonstrated that LSD, ergotamine, and other ergolines have functional selectivity for β-arrestin signaling at the 5-HT_2B_ while also being unbiased for the 5-HT_1B_ receptor (68). A comparison of crystal structures of LSD and ergotamine in both receptors revealed that the peptide appended to ergotamine was the cause of ergotamine’s superior biased signaling compared to LSD. An additional analysis of LSD complexed with 5-HT_2B_ by Wacker et al. in 2017, in combination with molecular dynamics simulations, revealed a structural basis for LSD’s slow binding kinetics at 5-HT_2A_ and 5-HT_2B_ (69). This work also revealed that the amide substituents in LSD play a significant role in the position of the ligand within the orthosteric pocket, the molecule’s long residence time, and its ability to promote β-arrestin translation. Similarly, solved crystal structures of ergotamine and ritanserin bound to 5-HT_2C_ by Peng et al. revealed distinctive binding modes for each compound and key receptor–ligand interactions that promote serotonin receptor-subtype selectivity (32). An analysis of serotonin, LSD, psilocin, and lisuride complexed with 5-HT_2A_ by Cao et al. displayed a secondary binding pose for psilocin and serotonin in which their indole cores interact with the extended binding pocket of the receptor (70). Their data suggest that serotonin/psilocin may adopt two different positions, either in the orthosteric binding pocket or the extended binding pocket, and these would produce different biological effects. This group campaigned to synthesize a molecule that would favor the extended binding pocket, with the hypothesis that this would promote β-arrestin recruitment bias at 5-HT_2A_. They successfully synthesized compounds IHCH-7079 and IHCH-7086 which displayed antidepressant effects without inducing hallucinogenic behavior in mouse models. Docking studies of tetrahydropyridines complexed with 5-HT_2A_ performed by Kaplan et al. led to the synthesis and further testing of 17 molecules (71). Among these, two displayed antagonist activity at 5-HT_2B_, one displayed antagonist activity at 5-HT_2A_, and another displayed agonist activity at 5-HT_2B_. Additional structure-based optimization led to the discovery of a selective 5-HT_2A_ antagonist and two 5-HT_2A_ partial agonists. The two agonists elicited antidepressant behavior in mouse models and low levels of head-twitch response when compared to LSD. Notably, these agonists were also quite selective and showed a 4.6–6.4-fold preference of 5-HT_2A_ over 5-HT_2B_ and a 29–51-fold preference over 5-HT_2C_. Later, Dong et al. described a fluorescent biosensor which was capable of predicting a compound’s hallucinogenic potential by detecting distinct GPCR conformations induced by hallucinogenic ligands *in vivo* (72). This biosensor was then used to identify a nonhallucinogenic DMT analog with neuroplasticity-promoting and antidepressant effects. Finally, 2-Br-LSD was demonstrated by Lewis et al. to be a non-hallucinogenic analog of LSD with neuroplasticity-promoting effects (31). Pharmacological profiling showed that 2-Br-LSD is a significantly more selective ligand than LSD and does not show activity at aminergic GPCRs known to affect blood pressure, heart rate, and other autonomic functions (e.g., 5-HT_2B_). Interestingly, while the compound was non-hallucinogenic, it still displayed agonism at 5-HT_2A_. However, the authors had shown that while 2-Br-LSD has high potency at 5-HT_2A_ (EC_50_ = 0.81 nM), it only partially activates the receptor (E_MAX_ = 60%, relative to serotonin); whereas LSD is nearly a full agonist (E_MAX_ = 92%, relative to serotonin). They later tested 2-Br-LSD as an antagonist in 5-HT_2A_ G_q_ dissociation and β-arrestin2 recruitment assays, and the analog did indeed act as a potent partial antagonist.

**Figure 2:**
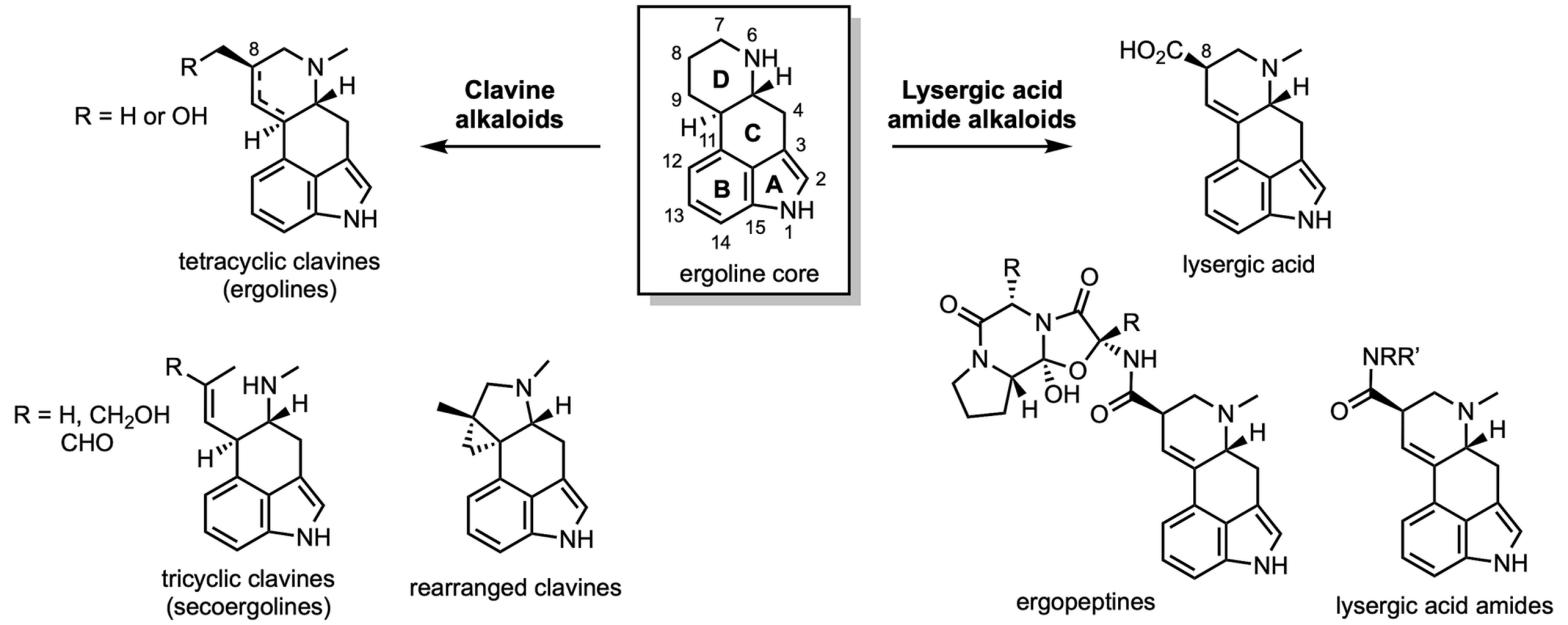
Structural overview of ergot and clavine alkaloids.

Many studies of psychedelics and their analogs focus on lysergic acid amides and dimethyltryptamine derivatives and their biological effects. Clavine alkaloids differ from lysergic acid derivatives by the structure of the quinoline ring system fused onto the indole core and the absence of a carboxylic acid functional group at C8 (Figure 2). They often have a greater conformational flexibility and are therefore more tunable receptor ligands (73, 74). However, clavine alkaloids are still comparatively understudied despite their potential for high bioactivity and superior receptor selectivity. As testimony to their medicinal effectiveness, several semisynthetic ergolines and clavines have reached the market for treating disorders and diseases such as migraines, cancer, Parkinson’s disease, and dementia (75, 76). For example, lisuride is structurally similar to LSD, yet it does not typically produce hallucinogenic effects and has often been used to treat Parkinson’s disease and migraines (77). It also produces antidepressant behavior in mice (78). Lisuride displays nanomolar binding affinity for most aminergic GPCRs including serotoninergic, adrenergic, and especially dopaminergic receptors. Its ability to treat Parkinson’s disease is believed to primarily stem from its agonist activity at the dopamine receptors (79). While lisuride has potent agonist activity at 5-HT_1A_ (80), it is a strong antagonist at 5-HT_2B_ and thus devoid of cardiac valvulopathy side effects (81). At 5-HT_2A_, lisuride is a G-protein biased agonist and this may be the mechanism responsible for how the medicinal properties of lisuride and potential hallucinogenic side effects are decoupled (78). However, strong activation of 5-HT_1A_ has been shown to block the head-twitch response in mice treated with a 5-HT_2A_/5-HT_2C_ agonist (82). Dopamine and adrenoreceptors may also play a significant role in modulating head twitch response. Bromocriptine, a 2-brominated derivative of ergocriptine is a medicinally relevant ergot alkaloid that has been used in combination with levodopa in the treatment of Parkinson’s disease (83). Bromocriptine has also been implicated as a potential therapeutic agent for type 2 diabetes (84). Similar to lisuride, it is a potent D_2_ agonist and has good binding affinity for a number of serotonin receptors. However, bromocriptine is an antagonist at D_1_, an inverse agonist at D_4_, and a partial agonist at 5-HT_2B_ (80). It also behaves as an agonist at 5-HT_2A_ and 5-HT_2C_ receptors and, interestingly, acts as an antagonist at the adrenoreceptors *α*_2A_, *α*_2B_, and *α*_2C_. Nicergoline has been used for the treatment of dementia as well as cerebrovascular, peripheral vascular, and balance disorders (85, 86). The compound has high binding affinity for *α*_1_ and 5-HT_1A_ receptors and demonstrated antagonism at these receptors (87). While nicergoline only has appreciable affinity for *α*_2_ and 5-HT_2_ receptors, it has low affinity for the dopamine receptors D_1_ and D_2_ and the muscarinic receptors M_1_ and M_2_. As a final example, ergotamine (*vida supra*) has been used for the prevention and treatment of migraines (88). It should be noted that the toxicity of these compounds and of all members of the ergot alkaloid class is a potential concern (89–92). Ergotism and ischemia can occur if the levels of ergot alkaloids in the body exceed a certain threshold. This typically occurs if a patient is co-treated with a cytochrome P450 inhibitor and the ergot alkaloid medication is not properly metabolized; therefore, prescribed dosages of ergot alkaloids are typically low (93). Potential toxicity should not discourage the development of these compounds into marketable drugs, but rather demonstrates the need for additional clinical and preclinical studies and investigations of natural and synthetic alkaloids before their full therapeutic potential can be unlocked.

## 2 Notable advancements

Most ergot alkaloid drugs are produced semi-synthetically from field cultivations or fermentations of ergot fungi (94). The clinically useful natural product precursors for derivatization are elymoclavine, lysergol, and lysergic acid, which can be isolated directly from the fungus or received from global saponification or reduction of the bulk mass of ergopeptines and lysergic acid amides. Recent studies to reconstitute the biosynthetic pathways of ergot alkaloids provide scalable access to single molecules. The biosynthesis of cycloclavine, a complex rearranged clavine alkaloid containing a cyclopropyl ring has been elucidated, and production of cycloclavine through heterologous expression in yeast, delivered titers of >500 mg L^−1^ (95). Microbial cell factories and cell-free systems, two unrelated systems for the biosynthesis of biomolecules, were combined to overproduce agroclavine in titers of 1,209 mg L^−1^ (96). The clavine oxidase (CloA) is a putative cytochrome P450 that catalyzes formation of lysergic acid from agroclavine in the ergot alkaloid biosynthetic pathway. A specific region between the F-G helices of CloA was identified as responsible for detecting and directing oxidation of agroclavine. An engineered, chimeric AT5 9Hypo CloA, which contained a clear substrate access channel and an enzyme surface cleft, increased levels of lysergic acid by 15-fold compared to the wildtype enzyme. Further discussion on scalable biosynthetic processes is beyond the scope of this review but remains an active field that holds potential to facilitate clavine and ergot alkaloid diversification. The biological implications of clavine alkaloids, closely related to LSD, have typically been evaluated through derivatizing natural products or investigations of readily derived stereoisomers.

### 2.1 Natural and substituted clavine alkaloids

Preliminary investigations into the SAR of the clavine alkaloids by Hofmann and Pertz established primary modalities pertinent for activity at CNS receptors. Notably, therapeutic investigations of clavine alkaloid derivatives were pioneered by Hofmann after his historic discovery of LSD (97). (+)-1-Methyl lysergol showed a 13-fold increase in serotonin inhibitory effect compared to naturally occurring (+)-lysergol, but 1-methyl-ergotamine was less potent than ergotamine, indicating this slight modification was mainly relevant to clavine alkaloids. Moreover, (+)-lysergol produces a pyretogenic effect, but the methylated analog initiates an inverse reaction and lowers the body temperature in rabbits. Finally, (+)-1-methyl lysergol (LD_50_ = 18 mg/kg) was over 50-fold less toxic than (+)-lysergol (LD_50_ = 0.32 mg/kg).

Furthermore, Pertz et al. investigated eight naturally occurring clavine alkaloids for antagonism/partial agonism at 5-HT_2A_ receptors and antagonism at *α*_1_-adrenoreceptors in blood vessels of rat tail arteries and aorta (73). While CNS receptor structure differs between species, they demonstrated that clavine alkaloids can have different modes of action compared to 5-HT. This concept was later supported by cryo-EM structures of LSD-bound 5-HT_2A_; the binding modes of the semi-synthetic ergot alkaloid differed from the agonist 25CN-NBOH (98). Clavines, with the exception of costaclavine containing the deactivating *cis*-CD ring junction, show moderate affinity to 5-HT_2A_ receptors and *α*_1_-adrenoreceptors. They display partial agonism activity by themselves, and some are antagonists of 5-HT at micromolar concentrations. Moreover, *N*1-isopropylation can increase affinity and specificity for 5-HT_2A_ vs. *α*_1_, which could improve the noxious effects associated with ergot alkaloids. In fact, a similar modification was previously demonstrated by Hofmann (*vide supra*). In a subsequent report, Pertz et al. investigated cycloalkanecarboxylic esters derived from lysergol, dihydrolysergol-I, and elymoclavine (74). As with the naturally occurring clavines, these derivatives displayed partial agonism and antagonism effects of the contractile properties of 5-HT. Thereby, SAR investigations into acyl group variation and *N*1-modification demonstrated that the tetracyclic indolo[4,3-*fg*]quinoline system was the primary pharmacophore, and hydroxy acylation with cycloalkyl groups coupled with *N*1-alkylation synergistically increased 5-HT_2A_ receptor selectivity over the α_1_-adrenoreceptor.

In 2017, Luo et al. disclosed an elegant asymmetric synthesis of (+)-lysergol, (+)-isolysergol, (−)-isolysergol, and (+)-13-fluorolysergol featuring a Ciamician-Dennstedt-type rearrangement to form the piperidine C ring and a Rh-carbenoid formal [3 + 2] cycloaddition to construct the ergoline scaffold (99). These analogs were subjected to agonism assays at four serotonin receptors, 5-HT_1A,2A–C_. CHO-K1 cells expressing each receptor were loaded with a dye and analyzed by FLIPR during addition of compounds or control agonist. All of the compounds displayed a reduced maximal activation for each receptor compared to 5-HT (see Table 1). For instance, while (+)-lysergol activated 5-HT_2A_ with an EC_50_ value of 1.6 nM, it was limited to 51% of the maximum 5-HT stimulation. Similarly, (+)-lysergol activated 5-HT_2C_ to 43% maximum of 5-HT stimulation. The unnatural substituted analog (+)-13-fluorolysergol, while selective for 5-HT_1A_ and 5-HT_2A_, only showed 17% maximal activation of 5-HT_2A_ compared to 5-HT. While less effective than the natural ligand, 5-HT, partial agonism and receptor selectivity garnered by ring substitution could lead to effective therapeutic agents without deleterious hallucinogenic effects (*vide supra*).

**Table 1:** Lysergol analog activation of 5-HT receptors reported by Luo et al. (99).

Entry	Compound	5-HT_1A_ (nM)	5-HT_2A_ (nM)	5-HT_2B_ (nM)	5-HT_2C_ (nM)
1	5-HT	11 ± 2	0.96 ± 0.14	0.071 ± 0.010	0.14 ± 0.03
2	(+)-lysergol	73 ± 6	1.6 ± 0.5^a^	>10,000	6.6 ± 1.4^b^
3	(±)-lysergine	342 ± 23	2.7 ± 1.6^c^	145 ± 54^d^	103 ± 9^e^
4	(−)-isolysergol	481 ± 31	>10,000	>10,000	>10,000
5	(+)-13-fluorolysergol	424 ± 27	12 ± 3^f^	>10,000	>10,000

a 51% max 5-HT stimulation.

b 43% max 5-HT stimulation.

c 57% max 5-HT stimulation.

d 36% max 5-HT stimulation.

e 42% max 5-HT stimulation.

f 17% max 5-HT stimulation.

### 2.2 Clavine alkaloid stereoisomers

Wipf et al. reported the shortest synthesis of racemic lysergols to date, and after chiral resolution of the enantiomers, investigated the effects of all four stereoisomers on several serotonin receptors. Of note, binding data of (−)-lysergol were disclosed for the first time. All but (−)-isolysergol maintained excellent affinity for 5-HT_1A,2A–C_ (100). Agonism effects of (+)-lysergol, (−)-lysergol, and (+)-isolysergol were calculated as a percent of control response to a known reference (Table 2). The agonism activity of (+)-lysergol corroborated previous investigations (100). With 5-fold reduced activity at 5-HT_2A_, the naturally occurring (+)-lysergol should have reduced psychoactive effects compared to LSD. Moreover, it displayed a similar profile to psilocin with about half of psilocin’s activity at 5-HT_2A_. Interestingly, (−)-lysergol was a selective agonist for 5-HT_2C_. Many 5-HT_2C_ activators that are used to regulate mood are poorly selective between 5-HT_2B_ and 5-HT_2A_, so further investigations and SAR would be justified to elaborate upon these findings. (+)-Isolysergol displayed very similar activation of 5-HT_1A_ compared to (+)-lysergol, but was less active towards 5-HT_2C_, suggesting the configuration of C(8) plays a more significant role in agonism activity towards 5-HT_2C_ than 5-HT_1A_.

**Table 2:** Clavine alkaloid activation of 5-HT receptors.

Compound	5-HT_1A_R	5-HT_2A_R	5-HT_2B_R	5-HT_2C_R
(+)-lysergol^a^	76.5%^b^	50.4%^c^	–	61%^d^
(−)-lysergol^a^	–	–	–	70%^e^
(+)-isolysergol^a^	52.2%^b^	–	–	78.5%^b^
(+)-cycloclavine (μM)	0.14	~10	>20	0.016
(−)-cycloclavine (μM)	~5	>50	>20	3.2
(−)-rugulovasine A (nM)	37	n.t.^f^	n.t.	n.t.
(+)-rugulovasine A (nM)	47	n.t.	n.t.	n.t.
(+)-rugulovasine B (nM)	<2	46	58	339
(−)-rugulovasine B (nM)	116	n.t.	n.t.	n.t.
DMT^g^ (μM)	0.075^h^	0.076	3.4	0.424^h^
Psilocin^g^ (μM)	0.123^h^	0.721^h^	>20	0.094^h^
D-LSD^g^ (μM)	0.003^h^	0.261^h^	12^i^	0.015^h^

a Cellular agonist effect was calculated as a % of control response to a known reference agonist for each target. CNS receptor assays were performed in singlicate at human receptors at Eurofins Cerep Panlabs. “–” indicates no agonism effects were detected at the tested concentrations.

b 250 nM.

c 1.3 μM.

d 10 nM.

e 2.0 μM.

f n.t. denotes “not tested.”

g Data taken from ref. 30.

h Inhibition constants Ki.

i EC50 values.

Interestingly, while (+)-lysergol had a high affinity for 5-HT_2B_, it produced no agonism effects within the tested range (101). Similarly, (+)-isolysergol showed high affinities for 5-HT_2A_ and 5-HT_2B_ without agonism effects. Since these ligands bind without inducing activation, further studies are required to determine if they act as antagonists, as findings from Pertz (*vide supra*) (73) suggest these clavines could also produce antagonism effects at other CNS receptors. Of particular note is also the stereospecificity of the binding/agonistic effects of the four lysergol stereoisomers. While not necessarily unexpected for rigid and selective compounds, this nonetheless highlights opportunities for future ligand designs at 5-HTRs.

Recently, investigations into the activation effects of rearranged clavine alkaloids, including (+)- and (−)-cycloclavine and all stereoisomers of rugulovasine have been reported (99, 100, 102, 103). Wipf et al. developed an asymmetric synthesis of cycloclavine and investigated the specific binding and activation of both natural and unnatural enantiomers at several CNS receptors (104). The naturally occurring (+)-cycloclavine was an agonist for 5-HT_1A_ (EC_50_ = 0.14 μM) and 5-HT_2C_ (EC_50_ = 0.016 μM). (−)-Cycloclavine displayed comparably much smaller activation effects, and only nominally affected 5-HT_2C_ (EC_50_ = 3.2 μM). (+)-Cycloclavine displayed a remarkably similar profile to (+)-lysergol, albeit with ~8-fold lower 5-HT_2A_ activation. Since both cycloclavine enantiomers were poor activators of 5-HT_2A_, they may only exhibit weak hallucinogenic effects, but (+)-cycloclavine could display promising mood-regulating properties.

Piersanti et al. disclosed an asymmetric synthesis of all stereoisomers of rugulovasines and evaluated their CNS receptor activity (103). They were tested for specific binding at serotonin (5-HT_1A,2A,2C_), adrenergic (*α*_2_), and dopamine (D_1,2L,3_) receptors, and were found to be specific solely for the first. Of the four isomers, (+)-rugulovasine B was the overall best ligand and (−)-rugulovasine B displayed poor affinity for all but 5-HT_1A_. Moreover, among the targets that were investigated, the rugulovasines were selective for 5-HT receptors. Since all four isomers displayed a strong affinity for 5-HT_1A_, they were subjected to an activation assay for that target. Each isomer was a strong activator of this receptor with nanomolar potencies. (+)-Rugulovasine was the most potent agonist (<2 nM) and was surprisingly more active than LSD. This significant enantiospecificity and potency translated to other 5-HT receptors. Among the seven other serotonin receptors tested (5-HT_1A,2B–C,5A,6,7_), (+)-rugulovasine B displayed nanomolar activation for all but 5-HT_5A_ (13.7 μM), in contrast to LSD which only displayed low activation of 5-HT_2B_ (12.0 μM). (+)-Rugulovasine B is a potent activator of 5-HT_2A_ and 5-HT_2B_, suggesting hallucinogenic, cardiotoxic, and europhoric effects might occur in consumers. Accordingly, further (ant)agonism assays of dopamine and adrenergic receptors of the rugulovasines are required to validate their therapeutic potential. Hopefully, the expedient synthetic approach will enable future investigations of the biologically understudied rugulovasine scaffold.

## 3 Conclusion

The exploration of clavine alkaloids as therapeutic agents is of continued and significant interest. Early work focused on improving the potency and decreasing the toxicity of natural clavines through minor structural modifications, such as *N*1-alkylation or *O*-acylation (73, 74, 97). More recently, efficient total syntheses of clavine alkaloids have enabled systematic investigations of stereoisomers (100, 103, 104) and analogs with significant structural modifications (99). (+)-Lysergol, (+)-isolysergol, and (+)-cycloclavine thus far bear the most potential as non-hallucinogenic “psychedelic” agents. While more CNS receptor profiling and *in vivo* studies are required, they maintain promising 5-HT receptor profiles, as they poorly activate receptors that cause deleterious effects [5-HT_2A_ (hallucinations) and 5-HT_2B_ (cardiotoxicity)], but strongly activate the mood-regulators 5-HT_1A_ and 5-HT_2C_. Moreover, (+)-lysergol displays a very similar profile to the clinically studied psychedelic psilocin at the 5-HT_1A,2A–C_ receptors, and derivatization with deep-seated changes achievable through *de novo* synthesis could provide access to more selective ligands, as demonstrated by Luo et al. (99). The understudied rearranged clavine scaffolds, including cycloclavine and the rugulovasines, also demonstrate unique enantiospecific activation of the CNS receptors, similar to the lysergols. Some clavines such as (+)-rugulovasine B can be potent 5-HT_2A_ activators; however, they are relatively unselective agonists and could produce detrimental side effects. Future SAR campaigns on the clavine alkaloid scaffold are well justified by their known biological profile and are likely to discover a broad range of novel and potentially therapeutic analogs, paving the way to decouple the development of new psychedelics from the study of the known natural product isolates.

## Data availability statement

The original contributions presented in the study are included in the article/supplementary material, further inquiries can be directed to the corresponding author.

## Author contributions

NT: Writing – original draft, Writing – review & editing. EP: Writing – original draft, Writing – review & editing. PW: Conceptualization, Supervision, Writing – review & editing.
